# A world of traditional healing in the Global South

**DOI:** 10.1192/bji.2023.30

**Published:** 2023-11

**Authors:** David Skuse

**Affiliations:** Professor of Behavioural and Brain Sciences, Division of Population, Policy and Practice, UCL Great Ormond Street Institute of Child Health, London, UK. Email: d.skuse@ucl.ac.uk

**Keywords:** Traditional healing, Global South, evidence-based, cultural diversity, religious beliefs

This November 2023 issue represents the end of my tenure as Editor in Chief of *BJPsych International*. I have held this position for 10 years; before that I was Deputy Editor to the Founding Editor, Hamid Ghodse, since the inception of the journal. It has been a fascinating journey and I have enjoyed every minute. *International Psychiatry* was originally published by the Royal College of Psychiatrists. Subsequently, when the suggestion was made that Cambridge University Press should take over that responsibility, I was uncertain of the implications for our identity and growth. I need not have worried. The support we have received from CUP has been tremendous. There have been so many innovations over the past five years that have provided added value to our readers, listeners and viewers. I was keen to introduce podcasts, and videos that would better illustrate the work our contributors are doing around the world than print alone. CUP responded with enthusiasm, facilitating our use of media and providing us with a wonderful website.

In recent years, we have witnessed the introduction of a CiteScore that is steadily growing and we have high Altmetric scores for many of our articles. I would like to take this opportunity to encourage our readers around the world to submit to the journal, which makes no charge for publication. Recently, our submission rate has suffered somewhat from the rash of predatory journals which are targeted particularly at our colleagues in countries that are relatively underrepresented among the most competitive scientific publications. There is, we suspect, a tendency for some potential contributors to be tempted by less rigorous reviewing procedures than we employ, at a price.

## This issue

During my tenure, our mission has been primarily to promulgate examples of novel mental healthcare practice from countries where there is little or no formal psychiatric care available to most of the population. In many such regions, patients turn first to traditional healers or the Church. Among articles published in this issue, we find mention of these traditional pathways to care in Qatar (Mohammed et al),^1^ Oman (Al-Sinawi et al),^2^ Malawi (Kokota et al)^3^ and Kenya (Matoke).^4^ I was particularly struck by the revelation in the article on the management of psychosis in Malawi^3^ that up to 60% of caregivers of people with psychosis initially seek help for their relative from traditional healers. The authors emphasise that traditional and religious beliefs and practices continue to play a large role in the pathway to care. These systems are integral to the core values and belief systems of Malawian culture and ‘should not be ignored’.

The North East England South Asia Mental health Alliance (NEESAMA) is the subject of an article (Devgun et al)^5^ that describes a partnership with mental health organisations in Afghanistan, Bangladesh, India, Nepal, Pakistan and Sri Lanka. The authors opine that lessons learned from collaborating with those providing mental health services in the ‘Global South’ could lead to the delivery of more appropriate services to ethnic minorities in wealthier northern hemisphere countries too. Sensitivity to cultural beliefs is an especially important issue to consider when managing mental health problems among those caught up in the movement of traumatised refugees from south to north.

Although I am stepping down as Editor, I am hopeful that my successor will build on our initial reports on the role of traditional healers around the world, who have too often been ignored (or even suppressed) by those whose mental health training has been ‘evidence based’. A collaboration between traditional healing practitioners and psychiatrists seems to me to be essential in so many countries, not only in the Global South. We need to understand how best to encourage those afflicted by mental disorders in societies and cultures that do not conceptualise those disorders in the same way as we do to come forward for evidence-based treatments. Lydia Matoke reminds us of the Kenyan traditions of medical care that were lost when Britain colonised the country and endeavoured to eliminate what the colonisers regarded as ‘witchcraft’. In conclusion, I suggest we should be a little humble and recognise that although our Western education in medicine has brought about many benefits internationally in the practice of medicine and surgery, the mind remains mysterious and far less accessible to empirical research. Susceptibility to poor mental health is massively influenced by culture. Remember, the opportunity to explore this little-understood entity is what brought many of us into psychiatry in the first place.

## Data Availability

Data availability is not applicable to this article as no new data were created or analysed in this study.
